# From incidental cerebellar atrophy to progressive motor syndrome: a case within the spectrum of *ATXN2*-associated neurodegeneration

**DOI:** 10.1007/s10072-026-09404-7

**Published:** 2026-10-15

**Authors:** Xin Huang, Yan Ma, Xinran Ma, Kailin Xia, Dongsheng Fan

**Affiliations:** 1 Department of Neurology, Peking University Third Hospital, 49 North Garden Road, Haidian District, Beijing, 100191 China; 2 Key Laboratory for Neuroscience, National Health Commission/Ministry of Education, Peking University, Beijing, China; 3 Beijing Key Laboratory of Biomarker and Translational Research in Neurodegenerative Diseases, Beijing, China

**Keywords:** *ATXN2*, CAG repeat expansion, Cerebellar atrophy, Motor syndrome

## Abstract

**Background:**

Increasing evidence suggests that *ATXN2*-related disorders represent a clinical spectrum with variable presentations.

**Case presentation:**

We report a 70-year-old Chinese man carrying an *ATXN2 *allele with 34 CAG repeats (20/34 repeats). Cerebellar atrophy was incidentally noted on magnetic resonance imaging (MRI) more than ten years before symptom onset. He later developed bradykinesia and rigidity. After rapid weight loss, he developed a rapidly progressive motor syndrome with clinical upper and lower motor neuron features, followed by type II respiratory failure requiring noninvasive ventilation.

**Conclusions:**

These findings support the notion that *ATXN2*-associated neurodegeneration may represent a clinical continuum.

## Background

Ataxin-2 (ATXN2) is a polyglutamine protein that is highly expressed in the central nervous system (CNS) [1]. *ATXN2* CAG repeat expansions are associated with a broad spectrum of neurological phenotypes [2, 3]. Alleles in the fully expanded range are classically associated with spinocerebellar ataxia type 2 (SCA2) [4, 5]. Intermediate-length expansions (27–33 repeats) are recognized as genetic risk factors for amyotrophic lateral sclerosis (ALS) [2]. However, repeat length alone does not fully predict the clinical phenotype. Increasing evidence suggests that *ATXN2*-related disorders represent a continuous spectrum rather than separate clinical entities [6]. Here, we report a Chinese man with an *ATXN2* CAG repeat expansion (34/20 repeats) who developed cerebellar atrophy, cognitive impairment, and a progressive motor syndrome, accompanied by bradykinesia, rigidity, and tremor.

## Case presentation

The patient was a 70-year-old man with a history of hypertension, diabetes mellitus (DM), coronary artery disease, and hyperlipidemia. He was a former smoker and alcohol consumer who had recently quit both.

At the age of 53, brain MRI performed for transient dizziness incidentally showed cerebellar atrophy. No definite cerebellar signs were documented at that time, and the dizziness resolved spontaneously. Detailed clinical records were unavailable.

At 65 years of age, he developed progressive gait disturbance and bradykinesia. He had difficulty initiating gait and stopping while walking. His walking reportedly improved after treatment with levodopa and selegiline, although the exact dosage and magnitude of the treatment response were not systematically documented. At 67 years of age, he developed intermittent bilateral action tremor, which occurred with action or posture but was absent at rest and was more prominent on the left. No history of REM sleep behavior disorder was reported. DAT-SPECT and formal UPDRS/MDS-UPDRS assessment were not performed.

Eleven months before admission (June 2024), he started semaglutide therapy for diabetes, followed by rapid weight loss (about 15 kg over 4 months). Subsequently, he developed progressive axial weakness, particularly difficulty maintaining an upright posture for prolonged periods. His symptoms continued to worsen despite treatment for lumbar spinal disease.

Six months before admission, he developed dysarthria and left-sided weakness. He frequently coughed while drinking and reported reduced cough strength. Marked forward trunk flexion became evident while walking. Four months before admission, he developed weakness in the right upper limb, which led to hospital admission. After admission, arterial blood gas analysis confirmed type II respiratory failure, and long-term noninvasive ventilation was subsequently required. Pulmonary function testing could not be performed because of rapid clinical deterioration. Percutaneous endoscopic gastrostomy was performed during hospitalization. At 6-month follow-up, his condition remained relatively stable.

### Family history

**Fig. 1:**
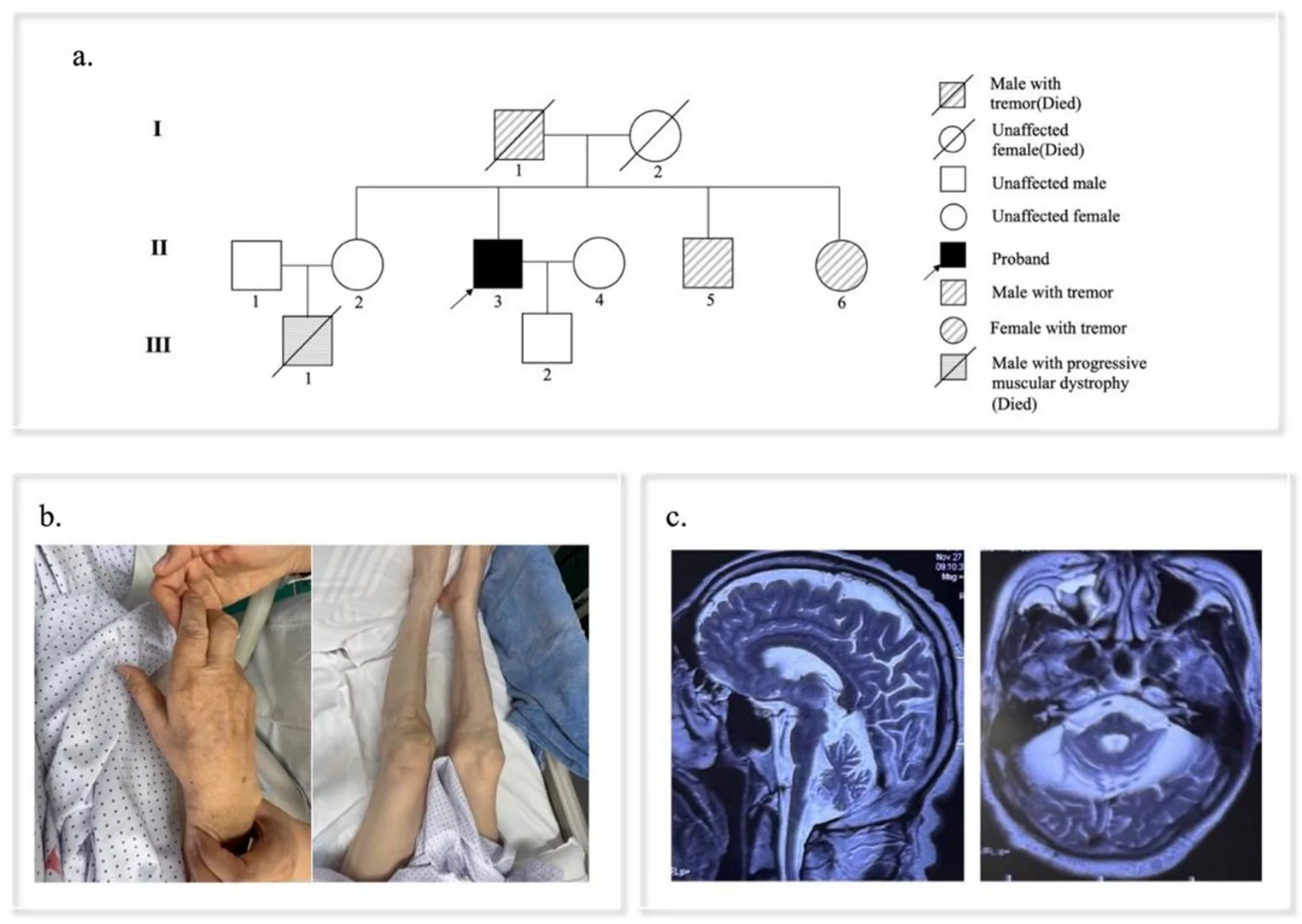
(**a**) The pedigree of the patient's family. The proband's father, brother(II-5), and sister(II-6) had involuntary tremors. The proband's nephew(III-1) died from progressive muscular dystrophy. (**b**) Pictures of the patient on admission. Left: Atrophy of the first dorsal interosseous muscle; Right: Atrophy of the legs. (**c**) Brain MRI of the patient in Sep. 2024

His father, brother, and sister reportedly had involuntary tremor (Fig. 1a). His brother is currently bedridden. A nephew (the son of his elder sister) died from progressive muscular dystrophy. However, genetic testing was not available for the affected relatives.

### Physical examination

On admission, the patient had reduced facial expression and dysarthria. There was no external ophthalmoplegia or nystagmus. Tongue atrophy and fasciculations were not observed, and the jaw jerk was not brisk.

Muscle atrophy was evident in the limbs, scapular region, and paraspinal area. Marked atrophy of the thenar and first dorsal interosseous muscles was observed, with relative preservation of the hypothenar muscles, consistent with a split-hand pattern. Weakness was more pronounced in the upper limbs and mild in the lower limbs (Fig. 1b). Axial tone was markedly increased, and tone in the right upper limb was slightly increased. Postural tremor was present in both hands.

Deep tendon reflexes were reduced in all four limbs. Bilateral Babinski signs were present. Hoffmann and Rossolimo signs were absent. Abdominal reflexes were absent bilaterally. Sensory examination was normal.

Clear cerebellar abnormalities were present, including impaired heel-to-shin performance and a broad-based gait. He had severe forward trunk flexion (approximately 70°). He was unable to walk independently, even with support. His gait was broad-based, with short steps and reduced arm swing.

There were no clinical features suggestive of autonomic dysfunction, including orthostatic hypotension or urinary incontinence.

### Laboratory and imaging findings

Immune and paraneoplastic evaluations were unremarkable. Serum neurofilament light chain (NfL) was elevated at 78.27 pg/mL (reference range, 8.00–62.00 pg/mL for individuals aged > 60 years). To differentiate peripheral neuropathy, antibodies associated with gangliosides, myasthenia gravis and node of Ranvier disorders were measured, revealing negative results.

Brain MRI showed atrophy involving the brainstem, cerebellum (including both vermis and hemispheres), and cerebral cortex (Fig. 1c).

Nerve conduction studies (NCS) showed predominantly motor abnormalities, particularly in the upper limbs, with additional sensory abnormalities. Lower-limb responses were relatively preserved. Needle electromyography (EMG) could not be completed because of respiratory failure. The NCS findings therefore could not determine whether the motor abnormalities were attributable to motor neuron degeneration or the concomitant sensorimotor polyneuropathy.

### Neuropsychological assessment and genetics

Neuropsychological assessment revealed significant cognitive impairment, with an MMSE score of 18, a MoCA score of 13, and an ECAS score of 67/136.

Next-generation sequencing identified no pathogenic variants in *SOD1* or other commonly recognized ALS-associated genes. No pathogenic repeat expansions were identified in *C9ORF72* or *NOTCH2NLC*. Repeat expansion analysis using PCR and fragment analysis identified a CAG repeat expansion in *ATXN2* (20/34 repeats).

## Discussion

This case illustrates the broad phenotypic spectrum of *ATXN2*-associated neurodegeneration, with cerebellar atrophy, cognitive decline, and a rapidly progressive motor syndrome occurring in the same patient, accompanied by bradykinesia, rigidity and tremor. Several clinical features suggested motor neuron involvement, including marked axial and limb weakness, muscle atrophy with a split-hand pattern, bilateral Babinski signs, dysarthria, dysphagia, reduced cough strength, and subsequent respiratory failure requiring noninvasive ventilation. The markedly elevated NfL level provided additional evidence of neuroaxonal injury. Although the patient exhibited clinical features suggestive of both upper and lower motor neuron involvement, the lower motor neuron component could not be reliably attributed to motor neuron degeneration because of concomitant sensorimotor polyneuropathy. In addition, needle EMG, which could have provided evidence of active and chronic denervation and helped distinguish motor neuron degeneration from peripheral neuropathy, could not be performed because of respiratory failure. Therefore, although the phenotype overlaps with ALS, the available evidence was insufficient to establish a diagnosis of definite or probable ALS according to the revised El Escorial criteria. The patient’s earlier cerebellar atrophy and subsequent progressive motor syndrome, together with the 34-repeat *ATXN2* allele, support an *ATXN2*-associated neurodegenerative phenotype. The 34-CAG allele is at the lower boundary of the reported expanded range associated with SCA2, although the phenotypic interpretation of an allele at this boundary may be influenced by repeat structure and other genetic or clinical factors. Repeat interruption analysis, including assessment of CAA interruptions, was not performed. Overlapping ataxia, parkinsonism and motor neuron phenotypes have been reported in patients with *ATXN2*-associated disease [7, 8]. The early presence of gait disturbance and bradykinesia followed by rapidly progressive motor deterioration further highlights the longitudinal evolution of the phenotype. Type II respiratory failure occurred in the context of progressive axial and bulbar weakness, suggesting a possible contribution from neuromuscular respiratory weakness, although this could not be objectively confirmed because pulmonary function and respiratory muscle assessments were unavailable. Overall, this case supports the concept that *ATXN2* repeat expansions may produce overlapping and evolving neurodegenerative phenotypes rather than a single clinical disorder.

## Conclusions

*ATXN2*-associated neurodegeneration should be considered in patients with overlapping cerebellar, cognitive, and motor manifestations. This case highlights the longitudinal evolution of the phenotype from cerebellar atrophy to a rapidly progressive motor syndrome with respiratory involvement. The absence of needle EMG, a formal SARA assessment and oculomotor assessment represent important limitations of this case.

## Data Availability

The information in this report is available from the corresponding author on reasonable request.
